# Functional Coding Variants in *SLC6A15*, a Possible Risk Gene for Major Depression

**DOI:** 10.1371/journal.pone.0068645

**Published:** 2013-07-16

**Authors:** Carina Quast, Serena Cuboni, Daniel Bader, André Altmann, Peter Weber, Janine Arloth, Simone Röh, Tanja Brückl, Marcus Ising, Anna Kopczak, Angelika Erhardt, Felix Hausch, Susanne Lucae, Elisabeth B. Binder

**Affiliations:** 1 Max Planck Institute of Psychiatry, Munich, Germany; 2 Department of Neurology and Neurological Sciences, Stanford University School of Medicine, Palo Alto, California, United States of America; University of Illinois at Chicago, United States of America

## Abstract

SLC6A15 is a neuron-specific neutral amino acid transporter that belongs to the solute carrier 6 gene family. This gene family is responsible for presynaptic re-uptake of the majority of neurotransmitters. Convergent data from human studies, animal models and pharmacological investigations suggest a possible role of SLC6A15 in major depressive disorder. In this work, we explored potential functional variants in this gene that could influence the activity of the amino acid transporter and thus downstream neuronal function and possibly the risk for stress-related psychiatric disorders. DNA from 400 depressed patients and 400 controls was screened for genetic variants using a pooled targeted re-sequencing approach. Results were verified by individual re-genotyping and validated non-synonymous coding variants were tested in an independent sample (N = 1934). Nine variants altering the amino acid sequence were then assessed for their functional effects by measuring SLC6A15 transporter activity in a cellular uptake assay. In total, we identified 405 genetic variants, including twelve non-synonymous variants. While none of the non-synonymous coding variants showed significant differences in case-control associations, two rare non-synonymous variants were associated with a significantly increased maximal ^3^H proline uptake as compared to the wildtype sequence. Our data suggest that genetic variants in the *SLC6A15* locus change the activity of the amino acid transporter and might thus influence its neuronal function and the risk for stress-related psychiatric disorders. As statistically significant association for rare variants might only be achieved in extremely large samples (N >70,000) functional exploration may shed light on putatively disease-relevant variants.

## Introduction

The *SLC6A15* gene encodes a sodium-dependent neutral amino acid transporter, belonging to the solute carrier 6 (*SLC6*) gene family that also includes the transporters for monoamines and gamma-amino butyric acid (GABA) [1]. This transporter is predominantly expressed in neurons with high levels in many regions of the brain including the hippocampus [2]. Proline is the amino acid with the highest affinity for *SLC6A15* and may serve as precursor for the synthesis of the neurotransmitter glutamate [1] and thus this transporter might be involved in the regulation of glutamate transmission [3]. Convergent data from human genetics, animal models and pharmacological studies suggest that *SLC6A15* may be involved in the pathophysiology of major depressive disorder (MDD).

In 2011, a genome-wide association study (GWAS) identified *SLC6A15* as a novel susceptibility gene for MDD [4]. The authors identified a single nucleotide polymorphism (SNP), rs1545843 about 690 kb downstream of *SLC6A15* on chr12q21.31, that was associated with unipolar depression at genome-wide significance (p = 1.41e-09) in a meta-analysis across seven samples. Expression quantitative trait locus data from lymphoblastoid cell lines as well as hippocampus showed that rs1545843 risk allele genotype status was associated with a decreased *SLC6A15* gene expression in both tissues. Furthermore the same common polymorphism showed an association with reduced hippocampal volume in patients with depression and reduced hippocampal neuronal integrity in healthy controls. The hippocampus is an important brain region modulating the hypothalamic-pituitary-adrenocortical (HPA) axis, which is dysregulated in depressed patients [5].

Schumacher *et al.* could show that rs1545843 risk allele genotype carriers have an enhanced adrenocorticotropic hormone (ACTH) and cortisol response in the combined dexamethasone/corticotrophin-releasing hormone (Dex/CRH) test. Furthermore, they observed an impaired memory and attention performance in risk genotype carriers also supporting a hippocampal dysfunction associated with this genetic variant [6].

Data from animal models also suggest that this gene plays an important role in stress-susceptibility. Behavioral phenotyping of *SLC6A15* knockout mice showed that these mice had increased levels of anxiety in the open field and dark box test immediately after they were subjected to a forced swim stress test as compared to wildtype mice, although this result could not be replicated in subsequent experiments [7]. The possibility of *SLC6A15* as a stress response gene was further supported by data from a chronic social stress model in outbred animals, were a reduced *SLC6A15* gene expression was observed in the hippocampus of stress-susceptible mice compared to stress-resilient mice [4].

Further indication for the possible involvement of *SLC6A15* in the pathophysiology of MDD originates from pharmacological investigations. The crystal structure of the bacterial leucine transporter (LeuT) derived from *Aquifex aeolicus* has been determined [8]. This transporter is a homolog of the human SLC6A15 transporter sharing 20–25% sequence identity and 40–45% sequence similarity. It has been shown that the LeuT transporter binds tricyclic antidepressant drugs at a site not overlapping with the leucine-binding site. This non-competitive binding prevents conformational changes of the protein and closes the molecular gate for leucine which inhibits the re-uptake of the substrate. As both the antidepressant- binding site and its inhibition mechanism of leucine uptake are probably conserved in humans one could assume that tricyclic antidepressant drugs also bind to the human transporter.

Given this convergent evidence for a possible role of *SLC6A15* in MDD, we aimed to further explore potential functional variants that could impact the amino acid transport of this protein and by extension neuronal function and possibly the risk for stress-related psychiatric disorders. To this end the *SLC6A15* gene locus was screened in depressed patients and controls for genetic variants using a pooled next-generation sequencing (NGS) approach. Following discovery, variants altering the amino acid sequence were then tested for their functional effects by measuring SLC6A15 transporter activity in a cellular uptake assay.

## Materials and Methods

### Ethics Statement

The current study was approved by the local ethics committee of the Ludwig-Maximilians-University (LMU) in Munich. Written informed consent was obtained from all individuals.

### Sample Characterization

#### Discovery sample

400 selected patients from the Munich Antidepressant Response Signature (MARS) project at the Max Planck Institute of Psychiatry (MPIP) in Munich were included in the study. Of the included patients, 88.0% suffered from recurrent depressive disorder, 12.0% presented a first depressive episode (see table 1 for demographic and clinical characteristics). Patients were recruited as described in Hennings *et al.* and Ising *et al.* [9], [10]. Severity of depression and anxiety was measured using the Hamilton Depression Scale (HAM-D) and the Hamilton Anxiety Scale (HAM-A) [11], [12]. Patients fulfilling the criteria of a HAM-D score ≥18 for recurrent depressive disorder or ≥20 for single depression episode at in-patient admission and an age at onset ≤55 were included in the study. Ethnicity was recorded using a self-report questionnaire for nationality, mother language and ethnicity of the subject itself and all four grandparents. All included patients were Caucasian, 78.0% of German origin.

**Table 1:** Demographic and clinical characteristics of the discovery and replication case-control sample.

Characteristics	Discovery sample		Replication sample	
	Patients	Controls	Patients	Controls
**N**	400	400	905	1029
**Sex**				
** male**	41.5% (166)	41.8% (167)	32.5% (294)	32.7% (336)
** female**	58.5% (234)	58.3% (233)	67.5% (611)	67.3% (693)
**Age (SD)**	46.9 (12.9)	46.9 (15.1)	51.1 (13.8)	50.7 (13.9
**Diagnosis**				
** recurrent depressive disorder**	88.0% (352)	–	100.0% (905)	–
** single depressive episode**	12.0% (48)	–	–	–
**HAM-D (SD)**	27.4 (5.0)	–	NA	–
**HAM-A (SD)**	25.5 (8.2)	–	NA	–
**age at onset (SD)**	31.9 (12.2)	–	36.0 (13.9)	–

HAM-D, Hamilton Depression Scale Score; HAM-A, Hamilton Anxiety Scale Score; SD, standard deviation; NA, not available.

400 controls from the general population were selected randomly and screened for the absence of psychiatric disorders as described in Erhardt *et al.* and Kohli *et al*, [13], [4]. The controls were matched to the patient sample for age, gender and ethnicity. All controls were Caucasian, 91.8% of German origin.

#### Replication sample

905 patients were recruited at the MPIP and psychiatric hospitals in Augsburg and Ingolstadt. All patients suffered from recurrent major depression (Table 1). For further details regarding patient recruitment see Lucae *et al.* and Muglia *et al.* [14], [15]. All included patients were Caucasian, 89.5% of German origin.

1029 controls, matched for age, gender and ethnicity to the patient sample, were selected randomly from a Munich-based community sample and screened for the absence of anxiety and affective disorders [14], [15]. All controls were Caucasian, 93.0% of German origin.

### DNA Amplification and Pooling Strategy

DNA was isolated from whole blood using a standardized extraction procedure (Puregene whole blood DNA-extraction kit, Gentra Systems Inc) and quantified using picogreen based fluorometry. Genomic DNA was combined in eight pools consisting of equimolar amounts of DNA from 50 depressed patients and eight pools consisting of equimolar amounts of DNA from 50 controls. In order to amplify the *SLC6A15* locus on chromosome 12, approximately 53 kb in length, eleven oligonucleotide primer pairs covering target regions between 2 and 11 kb were designed. Primers are listed in table S1. With exception of a 3.5 kb intronic region for which a working oligonucleotide primer could not be designed, the whole gene including the 5′ promoter region and 10 kb 3′ of the *SLC6A15* locus were covered. Individual Long Range PCR reactions were performed for each amplicon and each pool using LongAmp Taq DNA Polymerase (New England Biolabs (NEB)). For further information see Methods S1. Finally, four pools of PCR products consisting of equal amounts of amplified DNA from 100 patients and four pools of PCR products consisting of equal amounts of amplified DNA from 100 controls were used for NGS.

### Next-generation Sequencing

All eight DNA pools were prepared for the sequencing run performed on the Life Technologies SOLiD 4 sequencer following the manufacturer’s instructions for barcoded standard fragment library preparation. Bead production and enrichment was carried out using the EZ bead system. Each approximately 476 million beads were deposited on two full slides. The SOLiD sequencing run was performed using SOLiD ToP Fragment Barcoding Sequencing chemistry for a single F3 Tag with a read length of 50 bp.

Data analysis of this NGS run showed that the coverage of two amplicons was low in all eight sequenced libraries. Thus these two amplicons were re-sequenced in a second run. Barcoded fragment library and bead preparation were prepared as for the first run. Each approximately 139 million beads were deposited on two lanes of a flow chip for sequencing on the 5500xl SOLiD Sequencer using the SOLiD FWD SR sequencing chemistry for a single F3 Tag with a read length of 75 bp. The raw data of this experiment are deposited under the GenBank ID: SRP022550.

### Data Analysis of NGS Experiment: Variant Detection and Annotation

The raw reads of both SOLiD runs were subjected to the quality control (QC) procedure as described in Altmann *et al.* [16]. For further information regarding read numbers see table S2. The reads surviving the QC were aligned using BWA version 0.5.7 [17] and SHRiMP version 2.2.0 (Short Read Mapping Package) [18] to chromosome 12 of the human genome (NCBI36/hg18 for the first run and GRCh37/hg19 for the second run) allowing a maximum of four mismatches. Single nucleotide variant (SNV) calling and annotation steps were performed as described in Quast *et al.* [19] using vipR [16] and ANNOVAR [20]. Prerequisite for the inclusion into the variant calling procedure was that the coverage at a given base position in each pool had to be at least 5,000. Thus in the first run 81.4% of the whole sequence and 96.3% of the protein coding sequence were included into the SNV calling procedure. In the second run 74.3% of the whole sequence and 99.2% of the protein coding region had a mean coverage above 5,000. SNV calling was performed with QC filtered reads which were mapped using the BWA aligner and in a second approach using the SHRiMP aligner. Only genetic variants which were called in both approaches were included for further analysis while SNVs that were only detected in one alignment approach were excluded. We employed this mapping protocol in order to minimize the false discovery rate of rare SNVs.

### SNV Genotyping

Validation of 69 variants detected in the re-sequencing experiments was performed using MALDI-TOF (matrix-assisted laser desorption ionization time of flight) mass spectrometry on the Sequenom platform (San Diego, USA). We selected all coding variants and 53 variants across the whole minor allele frequency (MAF) range for technical validation. Correlations between the MAFs estimated by the Sequenom re-genotyping and the MAFs obtained by the NGS experiment were analysed using SPSS version 18.0.

Non-synonymous variants, which could be validated in the discovery sample, were re-genotyped in the replication sample using the same method as described above. Additionally, 22 non-synonymous SNVs from the Exome Sequencing Project (ESP) database (Exome Variant Server, http://evs.gs.washington.edu/EVS/), which currently incorporates 6,503 individuals with different ethnic background, were re-genotyped in the replication sample (accessed in October 2012).

Power calculations were performed using Quanto version 1.2.3 (http://hydra.usc.edu/gxe/) [21].

### Case-control Analysis

PLINK was used to test for case-control associations of common variants with a MAF >5% [22]. The cohort allelic sum test (CAST) was used to test the hypothesis that a combination of several rare variants is associated with a complex disease [23]. Specifically, we investigated the differences in the presence of minor alleles (PRA) and the sum of minor alleles (SMA) from a specific SNV set between depressed patients and controls. Our SNV set contained all discovered non-synonymous variants in the NGS experiment (N = 9). Statistical significance was assessed using independent samples t-test for the SRA and contingency tables for the PRA in SPSS version 18.0. P-values were not corrected for the multiple comparisons. The level of significance was set to 0.05.

### Experimental Functional Analysis

#### Site-directed mutagenesis

The cDNA of the long isoform of the human *SLC6A15* gene was inserted into the pEGFP-C1 vector (Clonetech). Variants of eGFP-h*SLC6A15* containing nine different non-synonymous SNVs were generated using site-directed mutagenesis using the primers listed in table S3. After amplification 1 µl of the restriction endonuclease DpnI (NEB) was added and the samples were incubated for 1 h at 37°C. 5 µl of each PCR product were transformed into E.*Coli* DH5α cells performing a heat shock for 1 minute at 42°C. Success of site-directed mutagenesis was verified by Sanger sequencing of all mutated plasmids.

#### SLC6A15 uptake assay

EGPF-h*SLC6A15* encoding plasmids (wildtype and nine mutants) were transfected into HEK293 cells using Lipofectamine (Invitrogen). HEK293 cells were cultured in Dulbecco’s Modified Eagle medium (DMEM, Gibco) containing 10% of fetal calf serum (FCS) and 5% Penicillin/Streptomycin at 37°C in a humidified incubator (5% CO_2_). The following day, transfected cells were detached, counted and plated in 96 well plates for the uptake measurement. The uptake of 20 nM ^3^H proline (Perkin Elmer) was measured in dependence of the concentration of the non-labeled amino acid L-proline (3 µM, 12 µM, 48 µM, 195 µM, 781 µM, 3.1 mM, 12.5 mM, 50.0 mM) using the Wallac MicroBeta luminescence counter (Perkin Elmer). The maximal uptake of ^3^H proline which occurs in the absence of antagonists and the IC_50_ were assessed using Sigma Plot. Differences in the mean ^3^H proline uptake between wildtype and mutant were assessed using general linear models in SPSS version 18.0. Transfection efficiency was included as covariate into the analysis. An alpha level of 0.05 after correction for multiple testing using the Bonferroni method was considered statistically significant.

#### Fluorescence imaging

HEK293 cells were plated on cover slips precoated with poli D-Lysine (PDL). After one day the medium was removed and cells were fixed with 4% paraformardehyde (PFA). Excess of PFA was removed performing two washing steps with PBS. The cover slips were mounted on slides using 4 µl mounting medium containing DAPI which stains cell nuclei. After few hours samples were analysed at the confocal microscope.

## Results

### Variant Identification Using NGS

In total, 405 genetic variants were detected in the sequencing runs. Of these, 218 (53.8%) have not been reported in the dbSNP137 database (accessed in April 2013). Furthermore, 225 (55.6%) variants have not been identified in the 1,000 Genome Project database (April 2012 release). As expected, more than 50% (N = 225) of the detected SNVs were rare with a MAF <0.5%, i.e., less than four occurrences among the 800 screened individuals.

16 variants (4.0%) were in the protein coding regions of the gene. Twelve of those 16 variants were non-synonymous and of those, seven have been previously reported in dbSNP137 and eight have been identified in the ESP database (accessed in April 2013). Three variants (0.7%) were in the 5′ untranslated region (UTR), 44 (10.9%) in the 3′ UTR and 257 (63.4%) in intronic sequences. 85 variants (21.0%) were 5′ or 3′ of the gene locus.

### Variant Validation Using Sequenom Re-genotyping

Using Sequenom re-genotyping as an independent method, 71.2% of the successfully re-genotyped variants (N = 66) could be confirmed as polymorphic (Figure S1), including nine non-synonymous coding SNVs, three of them only present in the short isoform of the *SLC6A15* locus (Table 2). For the validated variants, the correlation between the MAFs estimated from NGS and verified by re-genotyping was excellent with *r^2^ = 0.992.*

**Table 2:** Overview of all validated non-synonymous variants in the re-genotyping experiment combined with potential functional effects assessed by *in silico* analysis.

					Validation stage								NT conservation		AA conservation		
SNV	Basepositionon chr12	dbSNP137	Allele	initially found in	MAF Cases (%)	MAF Controls (%)	OR^1^	N^2^	mRNA isoform^3^	Location withingene	AA exchange	Splicing analysis^4^	PhastCons^5^	phyloP^6^	SIFT^7^	Panther^8^	Poly Phen2^9^
chr12_83809886	85285755	rs139354471	T>C	NGS	0.08	0.11	0.7	2734	long/short	exon 2	T49A	no effect	−	+	−	+	−
chr12_85277713	85277713	rs200478124	C>A	ESP	0.06	np	Case	1934	long/short	exon 5	K227N	ESE site broken	+	+	−	+	+
chr12_83801746	85277615	rs79063785	A>G	NGS	0.15	0.21	0.7	2734	short	exon 5	L260P	ESE site broken/newESS site	−	−	+	+	+
chr12_83801723	85277592	rs77477149	C>T	NGS	0.15	0.21	0.7	2734	short	exon 5	G268R	ESE site broken	−	−	−	−	−
chr12_83801692	85277561	rs17183577	T>A	NGS	17.50	19.25	0.9	800	short	exon 5	D278V	new ESE site	−	−	−	−	−
chr12_83790615	85266484	rs12424429	G>A	NGS	1.04	0.88	1.2	2734	long	exon 8	A400V	no effect	+	−	−	−	−
chr12_83790552	85266421	−	A>G	NGS	np	0.13	Con	800	long	exon 8	L421P	ESE site broken	+	+	+	−	−
chr12_83785100	85260969	rs201461650	A>G	NGS	0.08	0.07	1.1	2734	long	exon 10	I500T	ESE site broken/newESS site	+	−	−	+	−
chr12_85257265	85257265	rs138060449	T>C	ESP	0.22	0.10	2.3	1934	long	exon 11	N591D	no effect	+	+	−	+	−
chr12_85257235	85257235	−	C>T	ESP	np	0.05	Con	1934	long	exon 11	A601T	new ESS site	+	+	+	+	++
chr12_83779683	85255552	rs145111717	C>A	NGS	0.31	0.21	1.5	2734	long	exon12	E684D	no effect	+	−	+	−	++
chr12_83779607	85255476	rs144267969	C>T	NGS	0.13	np	Case	800	long	exon 12	G710R	no effect	+	+	+	−	++

SNV, single nucleotide variant; chr, chromosome; NGS, next-generation sequencing; ESP, Exome Sequencing Project; MAF, minor allele frequency; np, not polymorphic; OR, odds ratio; Con, control; N, number of individuals; AA, amino acid; ESE, exonic splicing enhancer; ESS, exonic splicing silencer; NT, nucleotide.

Location on chr. 12 is according to the February 2009 Human Reference Sequence (UCSC Genome Browser). Known SNVs are recorded in the dbSNP137 database.

1 Case: SNV only in cases; Con: SNV only in controls;

2 N = 2734, variant was present in both the discovery and the replication sample; N = 800, variant was only polymorphic in the discovery sample; N = 1934, variant was only polymorphic in the replication sample

3 Long isoform is according to the RefSeq annotation NM_182767, the short isoform NM_018057

4 FASTSNP;

5 ANNOVAR, phastCons 46-way alignment.

6 ANNOVAR, phyloP alignment, restricted to non-synonymous variants:+conserved (score >0.95), − non-conserved (score <0.95)

7 SIFT (sorting intolerant from tolerant): − tolerant (score >0.05),+possibly damaging (score <0.05)

8 Panther: − unlikely functional effect (pdeleterious <0.5),+possibly damaging (pdeleterious >0.5)

9 PolyPhen2: − benign (score <0.15),+possibly damaging (score 0.15–0.85),++probably damaging (score >0.85).

All nine non-synonymous SNVs validated in the discovery sample were re-genotyped in the replication sample and supplemented by 22 additional non-synonymous SNVs from the ESP that had not been detected in the discovery sample (Table S4). Only three of the ESP variants were polymorphic, two of them were only present in a single individual in the replication cohort (Table 2). For the validated non-synonymous variants (N = 12) which were also present in the ESP database (N = 11), the correlation between the MAFs denoted in the ESP for the European American population and obtained from the re-genotyping experiment in either the discovery, the replication or the combined sample was *r^2^ = 1.000.* For the non-synonymous variants genotyped in both cohorts, no consistent direction for over-representation in cases versus controls could be observed (Table S4).

### Annotation of Non-coding Variants

Mapping all 405 detected variants to ENCODE transcription factor binding sites (TFBSs) which were determined by Chip-Seq (http://genome.ucsc.edu/ENCODE/), we could identify 15 intronic and three intergenic variants in predicted TFBSs of different tissues including neuroblastoma cell lines (Table S5). In addition, these 18 variants were identified to be located in ENCODE/Duke DNaseI hypersensitivity sites in brain including cerebellum, frontal cerebrum and frontal cortex (http://genome.ucsc.edu/ENCODE/) [24]. Out of these variants with a potential influence on gene transcription the four variants with the highest/lowest OR were re-genotyped. Two variants which were previously reported in dbSNP137 could be validated in the discovery sample. Variants disrupting putative miRNA target sites in the 3′UTR of genes, predicted by TargetScanHuman 5.1 were not observed. Using PhastCons, we were able to identify four non-coding variants in conserved regions of the genome. These were re-genotyped in the discovery sample and one variant upstream and one variant in intron 1 of the gene could be validated, both already reported in dbSNP137. As the ORs of the above variants were around 1, they were not re-genotyped in the replication cohort.

### Case-control Analysis

None of the common variants (N = 62) showed a significant association with case-control status. Performing the CAST no significant differences in SRA and PRA between depressed patients (N = 1305) and controls (N = 1429) of the combined sample could be observed.

### Translation of the Detected Non-synonymous Variants into Function

#### Computational functional annotation

The potential functional effects of twelve non-synonymous coding variants were first investigated performing *in silico* analysis using SIFT [25], PolyPhen2 [26] and Panther [27] (Table 2). These three tools showed consistent predictions for only two non-synonymous variants to have a deleterious effect on protein function. Further evidence for possible functional consequences came from the evolutionary nucleotide conservation prediction tools PhastCons [28] and PhyloP [29] which identified five non-synonymous variants to be located in evolutionary conserved regions of the genome. Splicing analysis using FastSNP [30] predicted seven non-synonymous variants to create new exonic splicing enhancer (ESE) or silencer (ESS) motifs, or to disrupt already existing splicing motifs (Table 2).

#### Experimental functional analysis

**Figure 1:**
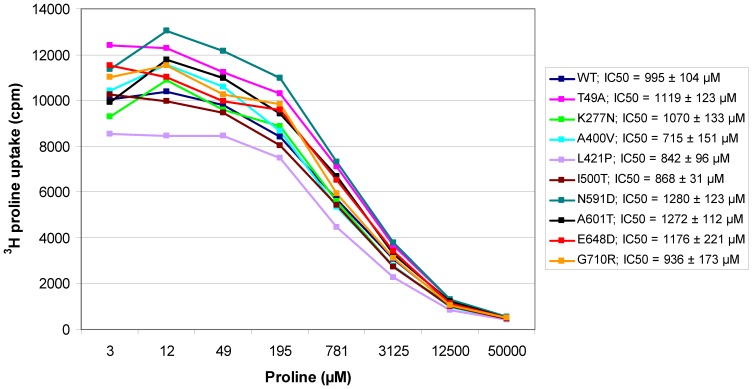
Inhibition of ^3^H proline transport by the non-radioactive labeled amino acid L-proline. Concentration of cold L-proline is plotted on the x-axis, ^3^H proline uptake as counts per minute (cpm) on the y-axis. Each datapoint represents the mean transport activity of triplicate samples.

The functional effects of all nine non-synonymous variants in the long human *SLC6A15* isoform (Table 3) were tested in a proline uptake assay. The IC_50_ values for ^3^H proline uptake did not differ between HEK cells transfected with plasmids containing the wildtype *SLC6A15* sequence and cells transfected with plasmids harbouring a point mutation in the *SLC6A15* gene (Figure 1).

**Table 3:** Overview of the mutant plasmids created by site-directed mutagenesis.

Mutant name	Nucleotide exchange	Amino acid exchange	Position in protein
hSLC6A15 T49A	A → G	Thr → Ala	49
hSLC6A15 K227N	G → C	Lys → Asn	227
hSLC6A15 A400V	C → T	Ala → Val	400
hSLC6A15 L421P	T → C	Leu → Pro	421
hSLC6A15 I500T	T → C	Ile → Thr	500
hSLC6A15 N591D	A → G	Asn → Asp	591
hSLC6A15 A601T	G → A	Ala → Thr	601
hSLC6A15 E684D	G → C	Glu → Asp	684
hSLC6A15 G710R	G → A	Gly → Arg	710

**Figure 2:**
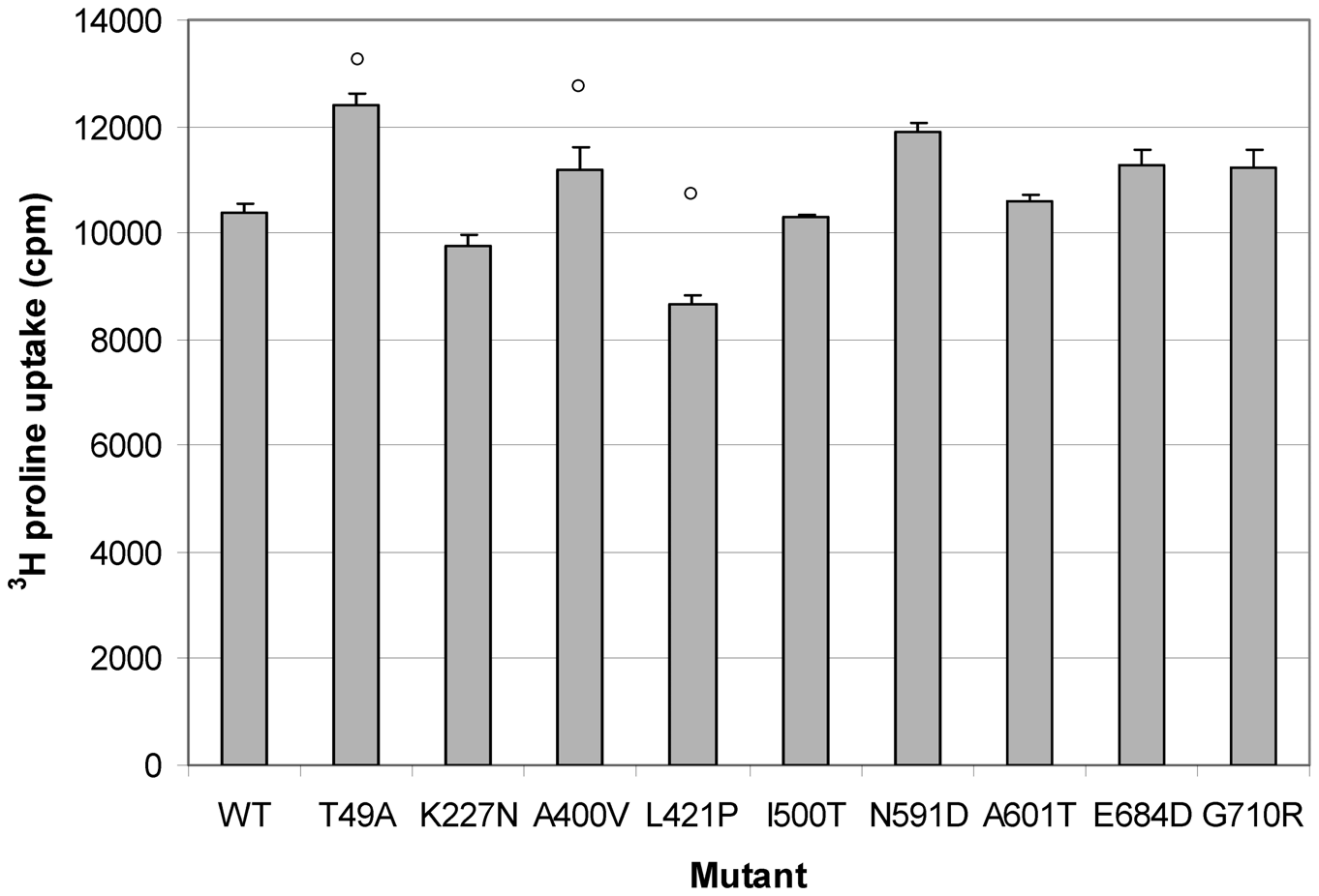
Maximal ^3^H proline uptake of wildtype (WT) and all tested mutants. The maximum in uptake was measured in the presence of 3 µM cold L-proline. Data are expressed as means ± standard deviation (SD) obtained from triplicate samples. Mutants with a circle were tested in a second independent experiment.

**Figure 3:**
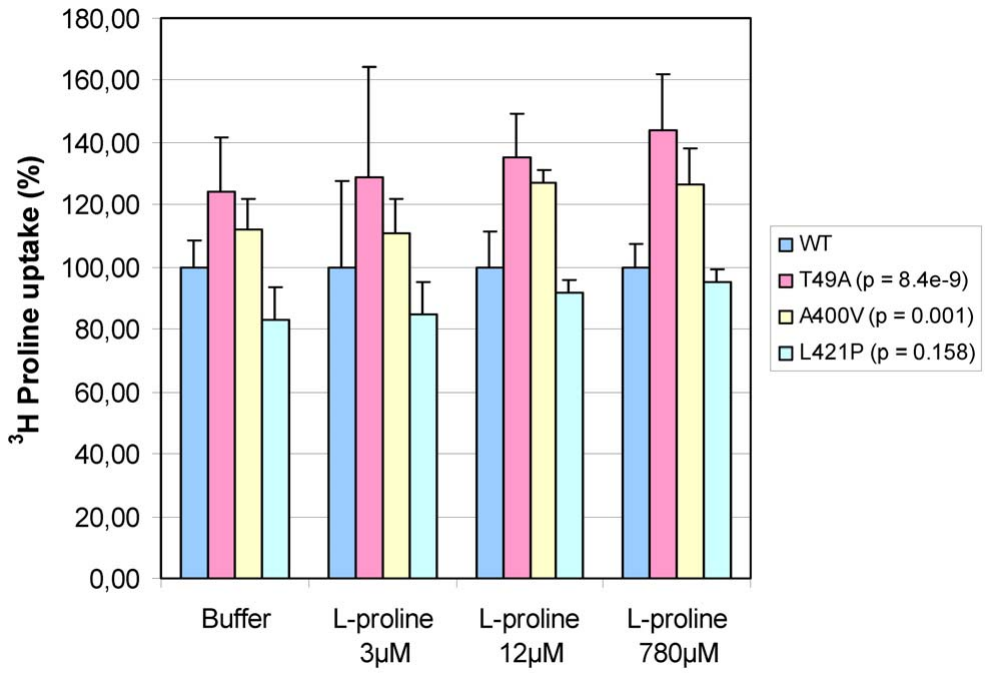
Repeated Bmax measurement of mutants that showed large differences in ^3^H proline uptake compared to WT. The uptake was measured under four different experimental conditions. Each bar represents the ^3^H proline uptake (mean ± SD) obtained from triplicates for the buffer solution, six samples for the 3 µM and 12 µM L-proline solution respectively and nine samples for the 780 µM L-proline solution. Corrected p-values given in brackets are based on the difference in mean ^3^H proline uptake between WT and tested mutant.

In contrast, the maximal uptake of ^3^H proline (Bmax) showed large differences, ranging from approximately 8600 to 12400 cpm (Figure 2). In order to verify these findings, the three mutants with the largest differences in ^3^H proline uptake compared to wildtype (T49A, A400V and L421P mutants) were selected and subjected to a second independent experiment. The results obtained in the first Bmax measurement could be replicated for all tested mutants (Figure 3). Across all concentrations of cold L-proline, significant differences in ^3^H proline uptake could be observed (p = 1.8e-7, in a two way ANOVA including mutant and cold proline concentration as the two predictors and transfection efficiency as a covariate (F = 18.9, df = 2). Mutant T49A and mutant A400V showed a significantly increased maximal ^3^H proline uptake compared to the wildtype, withstanding correction for multiple testing using the Bonferroni method (p = 8.4e-9 and p = 0.001 respectively). Mutant L421P showed a decrease in maximal uptake as in the first experiment, but this was not significant after adjustment for multiple comparisons (p nominal = 0.016, p corrected = 0.158).

**Figure 4:**
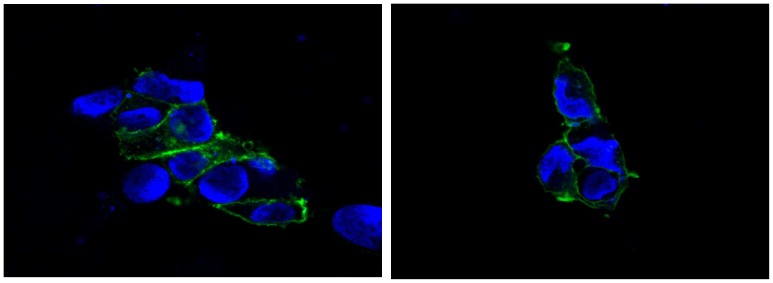
Cellular localization of the SLC6A15 protein. Both the wildtype cells (left) and the T49A mutant cells (right) showed a similar expression of SLC6A15 in the cell membrane. The localization of the eGFP-h*SLC6A15* fusion product is indicated in green. Cell nuclei were stained with DAPI (blue).

The cellular localization of the SLC6A15 transporter to the cell membrane was not changed in any of the mutants as indicated by fluorescence microscopy (Figure 4). In addition, these imaging experiments did not show any alterations in transporter levels at the cell membrane, indicating similar SLC6A15 expression levels in wildtype and mutants.

## Discussion

In this study we combined the detection of common and rare genetic variants in the *SLC6A15* gene using NGS with the functional characterization of these variants on the amino acid transporter activity using site-directed mutagenesis and cellular expression systems. While we could not confirm any significant differences in allele frequencies for any of the tested variants, we were able to identify two non-synonymous rare variants that lead to an increased transport of proline without changing the cellular localization of the SLC6A15 protein. This suggests that these rare coding variants may influence the biochemical function of this transporter and therefore the risk for major depression. However, extremely large case/control samples (over 70,000 for T49A and over 22,000 for A400V) would be required to detect the estimated ORs for these variants at an alpha level of 0.05 with a power of 0.8.

Since the establishment of high throughput sequencing methods, the identification of genetic variants, even those with a private character, has become a standard method in human genetics. While the detection of variants has become increasingly easy, their functional annotation remains a challenging task. Variants in protein coding regions of the genome resulting in amino acid substitutions, premature stop sites or deleted parts of a gene are the most obvious candidates for alterations of gene function. Indeed, such variants are heavily enriched among disease causing variation in rare/Mendelian disorders [31] while variants in regulatory regions seem to predominate as risk factors for common disorders [32]. However, it is likely that both common and rare variants contribute to the risk of common disorders [33], so that the exploration of both aspects will be important for a complete picture of disease risk. In this manuscript, we have focused on the functional characterization of non-synonymous coding variants in *SLC6A15*, a gene for which common risk regulatory variants have been identified.

The functional annotation of coding variants is of paramount importance as every individual carries 20,000–24,000 of such variants including 10,000–11,000 non-synonymous coding variants that could be deleterious for gene function, but in most cases are not [34]. Computational approaches may provide a fast and easy assessment of the functional relevance of such coding variants. A variety of tools has been developed based on the principle of sequence homology between organisms [35]. It is assumed that disease-causing variants are more likely at positions of the genome that are conserved and have not been removed by natural selection [36], [29]. One major disadvantage of these prediction methods is that direct comparisons of results obtained from different tools are problematic as each tool uses different algorithms and sequence databases as reference for the deleteriousness estimation. Some tools additionally include information about the structure of the protein into their predictions [37], [38]. Other tools incorporate biochemical data such as positions of active sites and disulfide bridges, charge of amino acids or protein-protein interactions [39]. The integration of structural and biochemical information to comparative sequence analysis could significantly improve predictions of deleteriousness [40], [41]. In this study, we used the three protein-sequence based prediction tools SIFT, PolyPhen2 and Panther and two nucleotide-sequence based prediction tools PhyloP and PhastCons. From our twelve detected non-synonymous variants only one was predicted to be functionally relevant in all tools (see table 2).

Although computational functional analyses are convenient, their inconsistent results indicate the need for experimental analysis. The prerequisite for experimental analyses is that the function of the gene product is known. While this point sounds trivial, a large number of human proteins are lacking sufficient functional annotation to design an experimental assay. In our case, it is known that SLC6A15 transports neutral amino acids into the cell, predominantly in neurons [1]. Proline is one substrate of the transporter and its uptake is a measurable property that associates with function. The two rare variants in the SLC6A15 protein T49A and A400V (Figure 3) were associated with a significant increase in maximal proline uptake in HEK cells. High levels of proline have been shown to be neurotoxic and have been associated with CNS symptoms such as seizures and mental retardation [42]. Furthermore, SLC6A15 is not only a transporter for proline but also transports other neutral amino acids like methionine and leucine. Methionine is a precursor of S-adenosylmethionine (SAM). This major methyl group donor in humans transfers methyl groups to different substrates including DNA nucleotides and histones. SAM metabolism has been associated with different diseases including psychiatric disorders such as MDD [43]. Leucine is a major donor of nitrogen for the synthesis of glutamate and GABA [44]. Therefore an alteration in the maximal amino acid uptake could have in impact on glutamatergic transmission which is connected to psychiatric disorders [45]. This hypothesis is further supported by a previously published study showing that SLC6A15 is expressed in glutamatergic and GABAergic neurons [46].

The observed alterations in amino acid uptake related to the coding variants might be explained by a number of mechanisms including altered maximal transporter velocity, but also altered expression, protein stability or membrane localization. The fact that fluorescence imaging showed similar protein levels in the plasma membrane, however, supports that functional rather than quantitative changes of the transporter may underly the observed genetic effects and that these rare non-synonymous variants indeed influence levels of amino acids in the cell and thus amino acid metabolism.

While the experimental analysis identified T49A and A400V to influence the levels of proline in HEK cells, for T49A only Panther and PhyloP and for A400V only PhastCons did predict an influence on protein function. This discrepancy is not surprising as the Encyclopedia of DNA Elements (ENCODE) project found that the correlation between estimates obtained from evolutionary annotations and estimates derived from experiments is only modest [24], [47]. One reason for this modest correlation might be that genetic variants that are biochemically functional do not necessarily have to be biologically relevant and may not affect the phenotype of interest [24], [48]. Indeed, even though the investigated variants in *SLC6A15* alter proline uptake it can not automatically be concluded that the altered amino acid levels are associated with an altered risk for the investigated phenotype MDD. While addressing the question of biological relevance for psychiatric disorders by genetic association studies may be difficult due to the extremely large samples sizes required (see above), additional experiments in neuronal cells lacking endogenous *SLC6A15* or humanized transgenic animals may shed more light on the putative relevance of these biochemical differences on biological measures and behavioral phenotypes. In addition, for stress-related psychiatric disorders such as MDD, the risk conveyed by a specific variant may only be unmasked with exposure to stress or trauma, so that a strict case/control design may be insufficient.

Newer data from exome re-sequencing projects indicated that rare variants might not contribute to disease risk with much higher ORs than common variants [unpublished data], so that the samples sizes needed to detect the significant association for any one of them may be prohibitive. One possible solution could be the use of burden testing, which assesses whether a combination of rare variants is non-randomly distributed between different groups. This approach may enhance the power to detect associations of multiple rare variants in specific candidate genes or pathways [49], but will still require large sample sizes comprising at least several thousand cases and controls to achieve genome-wide significance [50]. A drawback of burden testing is that functional relevant variants should be identified and analyzed together. This is typically done using *in silico* annotation, without proof of true biological relevance and variants with opposite effects can even combined into the same group. Experimental functional annotation would thus likely increase the power of such burden tests [51].

In conclusion, our study suggests that rare variants in *SLC6A15* may have an influence on the biochemical function of this amino acid transporter. To further evaluate the impact of these variants on neurobiological phenotypes and ultimately MDD, additional *in vitro* and *in vivo* experiments as well as very large samples sizes for case-control association or gene × environment interaction studies will be required. Our data also highlight that if possible, experimental validations should be performed to assess the functionality of coding variants as computational tools only give insufficient information.

## Supporting Information

Figure S1 **Overview of the SNV validation performing Sequenom re-genotyping.** Denoted MAF was estimated from NGS.
(DOC)

Table S1 **Oligonucleotides used for amplification of the** ***SLC6A15*** **locus via Long Range PCR.**
(DOC)

Table S2 **Number of reads obtained in the two sequencing runs.** Reads were mapped using the BWA aligner. All reads are given in millions.
(DOC)

Table S3 **Oligonucleotide primers used for site-directed mutagenesis.** The sequence encoding for the substituted amino acid is underlined. The changed nucleotide is bold.
(DOC)

Table S4 **Summary of the re-genotyping of all non-synonymous variants in the discovery sample, replication sample and combined sample.**
(DOC)

Table S5 **Overview of all ENCODE TFBSs, identified using Chip-Seq (chromatin immunoprecipitation with antibodies against the transcription factor and sequencing of the precipitated DNA).** TFBSs can overlap so that a variant can be located in two or more sites. All 18 variants were identified to be located in ENCODE/Duke DNaseI hypersensitivity sites in brain including cerebellum, frontal cerebrum and frontal cortex (http://genome.ucsc.edu/ENCODE/).
(DOC)

Methods S1 (DOC)
