# Supplementary material for: Functional Coding Variants in SLC6A15, a Possible Risk Gene for Major Depression
Source: PLoS One. 2013 Jul 16;8(7):e68645. doi: 10.1371/journal.pone.0068645 (PMC3712998; doi:10.1371/journal.pone.0068645)

**Figure S1**

Overview of the SNV validation performing Sequenom re-genotyping. Denoted MAF was estimated from NGS.


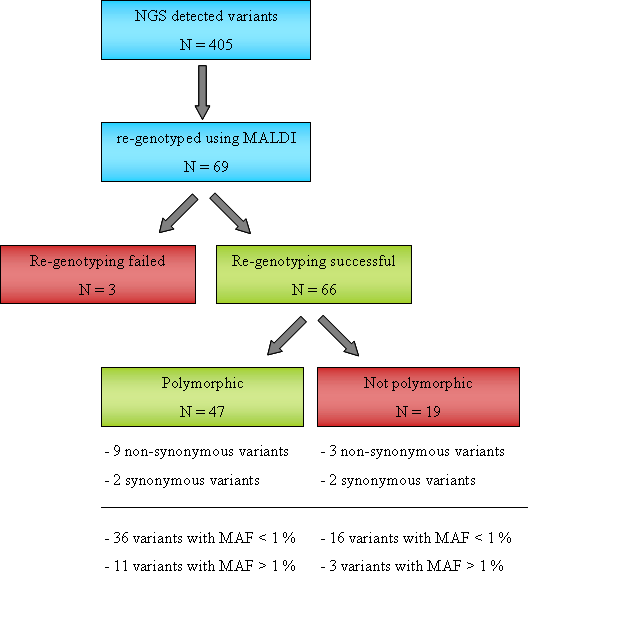

Supplement: Figure S1 — Overview of the SNV validation performing Sequenom re-genotyping. Denoted MAF was estimated from NGS. (DOC) [file pone.0068645.s001.doc]
