# Supplementary material for: Functional Coding Variants in SLC6A15, a Possible Risk Gene for Major Depression
Source: PLoS One. 2013 Jul 16;8(7):e68645. doi: 10.1371/journal.pone.0068645 (PMC3712998; doi:10.1371/journal.pone.0068645)
Supplement: Table S1 — Oligonucleotides used for amplification of the SLC6A15 locus via Long Range PCR. (DOC) [file pone.0068645.s002.doc]

**Table S1**

Oligonucleotides used for amplification of the *SLC6A15* locus via Long Range PCR.

| **Primer name** | **Primer sequence** | **Amplicon position in genome** | **Amplicon length (bp)** |
| --- | --- | --- | --- |
|  |  |  |  |
| 1fwd | TTTTCTCCCCACCAGCCCCCAATCTGCT | chr12:85302558-85313254 | 10697 |
| 1rev | AACAGCTGAGAAAGCCAGGCCCAAACATCA |  |  |
| 2fwd | AAAACGTCTGCTTCTCCTGCTAGAAACCCCA | chr12:85297924-85308787 | 10864 |
| 2rev | CTCCCACACCAATCCCATGTTGGCCATTTT |  |  |
| 3fwd | GTGATCTGTCAGTTCCAAGAAGGTGTT | chr12:85292757-85303571 | 10815 |
| 3rev | AAAAGAGAGCTTGGTGGCTATCAAAAG |  |  |
| 4fwd | AGCCCAAGAATTCCGCCCTTCATTTCTGGAA | chr12:85287367-85297848 | 10482 |
| 4rev | ACTGCTGCTACCTTCTGGTCAAAGCAAACCA |  |  |
| 5fwd | TGATTTGTGAGAAACAAAAAGCAGGAT | chr12:85284231-85293396 | 9166 |
| 5rev | GATGCTGGATAAGAGGCAAAGAAAAGT |  |  |
| 6fwd | CACAACTTGCAAATCCAATCCCGCCCAGTT | chr12:85279692-85289950 | 10259 |
| 6rev | TCTTCGGTGCAGATGAAGTGCAGTGAGTGAT |  |  |
| 7fwd | TGGGTTCCATGAGGACAGACTGTGGCCTATTA | chr12:85273578-85283585 | 10008 |
| 7rev | ACACTACCCATGTGACCTTTCACAGGCTACCT |  |  |
| 8fwd | AGGCAGCCGCCAGGAGTGACAAAGAAT | chr12:85269233-85271176 | 1944 |
| 8rev | AAACCAAGGGGCAGCCAGCAATTCAGTT |  |  |
| 9fwd | ACATATGCTCGGGGCAGAGCACAAACGTAA | chr12:85257053-85268042 | 10990 |
| 9rev | AGAGGACACGCCATTTGCCATTGTTTGCA |  |  |
| 10fwd | ATTTCCTTATCTGCCAAGTGAAACCAT | chr12:85252553-85263883 | 11331 |
| 10rev | TTCCTATCCAAAAAGTGCATAGCTGAA |  |  |
| 11fwd | AGGCACCACATGGCACGTTTTTGCTGT | chr12:85243329-85253392 | 10064 |
| 11rev | TCTTCTCTCACTCTCTTGCCATGGGGGC |  |  |
|  |  |  |  |

Amplicon position is according to the February 2009 Human Reference Sequence (UCSC Genome Browser).
