# Supplementary material for: Functional Coding Variants in SLC6A15, a Possible Risk Gene for Major Depression
Source: PLoS One. 2013 Jul 16;8(7):e68645. doi: 10.1371/journal.pone.0068645 (PMC3712998; doi:10.1371/journal.pone.0068645)
Supplement: Table S2 — Number of reads obtained in the two sequencing runs. Reads were mapped using the BWA aligner. All reads are given in millions. (DOC) [file pone.0068645.s003.doc]

**Table S2**

Number of reads obtained in the two sequencing runs. Reads were mapped using the BWA aligner. All reads are given in millions.

| **Pool** | **NGS Run 1** | | | **NGS Run 2** | | |
| --- | --- | --- | --- | --- | --- | --- |
|  | **raw reads** | **QC trimmed** | **mappable reads** | **raw reads** | **QC trimmed** | **mappable reads** |
|  |  |  |  |  |  |  |
| **Cases1** | 84.6 | 57.6 | 51.0 | 26.6 | 23.8 | 20.7 |
| **Cases2** | 83.6 | 56.0 | 46.8 | 33.1 | 29.6 | 25.7 |
| **Cases3** | 84.0 | 56.3 | 48.0 | 26.6 | 23.9 | 20.9 |
| **Cases4** | 90.8 | 59.0 | 49.3 | 42.9 | 38.4 | 33.6 |
| **Controls1** | 79.4 | 52.6 | 45.4 | 28.3 | 25.2 | 21.7 |
| **Controls2** | 96.4 | 62.6 | 54.1 | 23.0 | 20.6 | 17.8 |
| **Controls3** | 64.6 | 43.1 | 36.0 | 38.1 | 34.0 | 29.8 |
| **Controls4** | 85.3 | 56.7 | 47.2 | 34.1 | 30.5 | 26.5 |
|  |  |  |  |  |  |  |
| **Total** | 668.6 | 443.9 | 377.7 | 252.8 | 226.0 | 196.8 |

NGS, next-generation sequencing; QC, quality control.
