# Supplementary material for: Functional Coding Variants in SLC6A15, a Possible Risk Gene for Major Depression
Source: PLoS One. 2013 Jul 16;8(7):e68645. doi: 10.1371/journal.pone.0068645 (PMC3712998; doi:10.1371/journal.pone.0068645)
Supplement: Table S3 — Oligonucleotide primers used for site-directed mutagenesis. The sequence encoding for the substituted amino acid is underlined. The changed nucleotide is bold. (DOC) [file pone.0068645.s004.doc]

**Table S3**

Oligonucleotide primers used for site-directed mutagenesis. The sequence encoding for the substituted amino acid is denoted in italics. The changed nucleotide is bold.

| **Mutant** | **Primer name** | **Primer sequence** |
| --- | --- | --- |
|  |  |  |
| **hSLC6A15 T49A** | hSLC6A15 T49A-fwd | 5'-GGCCAGGAAGAGAAAGAT***G****CA*GATGTTGAAGAAGG-3' |
|  | hSLC6A15 T49A-rev | 5'-CCTTCTTCAACATC*TG****C***ATCTTTCTCTTCCTGGCC-3' |
| **hSLC6A15 K227N** | hSLC6A15 K227N-fwd | 5'-GGGGGCTTAAACTGG*AA****C***ATGACCATCTGCTTG-3' |
|  | hSLC6A15 K227N-rev | 5'-CAAGCAGATGGTCAT***G****TT*CCAGTTTAAGCCCC-3' |
| **hSLC6A15 A400V** | hSLC6A15 A400V-fwd | 5'-CAACCTTTCAACTGTTACT*G****T****A*GAAGATTATCATTTAGTTTATGAC-3' |
|  | hSLC6A15 A400V-rev | 5'-GTCATAAACTAAATGATAATCTTC*T****A****C*AGTAACAGTTGAAAGGTTG-3' |
| **hSLC6A15 L421P** | hSLC6A15 L421P-fwd | 5'-GAAGAGTTTCCTGCT*C****C****T*CATCTCAATTCCTGTAAAATTG-3' |
|  | hSLC6A15 L421P-rev | 5'-CAATTTTACAGGAATTGAGATG*A****G****G*AGCAGGAAACTCTTC-3' |
| **hSLC6A15 I500T** | hSLC6A15 I500T-fwd | 5'-GAGGAAAGAAATTCTTACTGTT*A****C****C*TGTTGTCTTCTGGC-3' |
|  | hSLC6A15 I500T-rev | 5'-GCCAGAAGACAACA*G****G****T*AACAGTAAGAATTTCTTTCCTC-3' |
| **hSLC6A15 N591D** | hSLC6A15 N591D-fwd | 5'-GCTAGTGTTGTG***G****AT*ATGGGATTAAGTCCTCCT-3' |
|  | hSLC6A15 N591D-rev | 5'-AGGAGGACTTAATCCCAT*AT****C***CACAACACTAGC-3' |
| **hSLC6A15 A601T** | hSLC6A15 A601T-fwd | 5'-CTCCTGGCTATAAC***A****CA*TGGATTGAAGATAAGG-3' |
|  | hSLC6A15 A601T-rev | 5'-CCTTATCTTCAATCCA*TG****T***GTTATAGCCAGGAG-3' |
| **hSLC6A15 E684D** | hSLC6A15 E684D-fwd | 5'-GGAAAAATACCGAGC*GA****C***ATGCCATCTCCAAATTTTG-3' |
|  | hSLC6A15 E684D-rev | 5'-CAAAATTTGGAGATGGCAT***G****TC*GCTCGGTATTTTTCC-3' |
| **hSLC6A15 G710R** | hSLC6A15 G710R-fwd | 5'-GGATACTGCTCCCAAT***A****GA*CGGTATGGAATAGG-3' |
|  | hSLC6A15 G710R-rev | 5'-CCTATTCCATACCG*TC****T***ATTGGGAGCAGTATCC-3' |
|  |  |  |
