# Supplementary material for: Functional Coding Variants in SLC6A15, a Possible Risk Gene for Major Depression
Source: PLoS One. 2013 Jul 16;8(7):e68645. doi: 10.1371/journal.pone.0068645 (PMC3712998; doi:10.1371/journal.pone.0068645)
Supplement: Table S4 — Summary of the re-genotyping of all non-synonymous variants in the discovery sample, replication sample and combined sample. (DOC) [file pone.0068645.s005.doc]

**Table S4**

Summary of the re-genotyping of all non-synonymous variants in the discovery sample, replication sample and combined sample.

|  |  |  |  |  |  |  | **Discovery sample**  **(N = 800)** | | | **Replication sample**  **(N = 1934)** | | | **Combined sample**  **(N = 2734)** | | |
| --- | --- | --- | --- | --- | --- | --- | --- | --- | --- | --- | --- | --- | --- | --- | --- |
| **SNV** | **Base position on chr.12** | **dbSNP137** | **mRNA Isoform^1^** | **Location within gene** | **AA exchange** | **initially found in** | **MAF Cases (%)** | **MAF Controls (%)** | **OR** | **MAF Cases (%)** | **MAF Controls (%)** | **OR** | **MAF Cases (%)** | **MAF Controls (%)** | **OR** |
| chr12_85285806 | 85285806 | rs150047699 | long / short | exon 2 | D32H | ESP | not re-genotyped | | | np | np |  |  |  |  |
| chr12_83809886 | 85285755 | rs139354471 | long / short | exon 2 | T49A | NGS | 0.13 | 0.13 | 1.0 | 0.06 | 0.10 | 0.6 | 0.08 | 0.11 | 0.7 |
| chr12_85285676 | 85285676 | rs150860765 | long / short | exon 2 | V75A | ESP | not re-genotyped | | | np | np |  |  |  |  |
| chr12_85279737 | 85279737 | - | long / short | exon 3 | N134D | ESP | not re-genotyped | | | np | np |  |  |  |  |
| chr12_85277713 | 85277713 | rs200478124 | long /short | exon 5 | K227N | ESP | not re-genotyped | | | 0.06 | np |  |  |  |  |
| chr12_85277622 | 85277622 | rs142725706 | short | exon 5 | P258S | ESP | not re-genotyped | | | np | np |  |  |  |  |
| chr12_83801746 | 85277615 | rs79063785 | short | exon 5 | L260P | NGS | 0.25 | 0.13 | 2.0 | 0.11 | 0.24 | 0.5 | 0.15 | 0.21 | 0.7 |
| chr12_83801723 | 85277592 | rs77477149 | short | exon 5 | G268R | NGS | 0.25 | 0.13 | 2.0 | 0.11 | 0.24 | 0.5 | 0.15 | 0.21 | 0.7 |
| chr12_85277576 | 85277576 | - | short | exon 5 | S273L | ESP | not re-genotyped | | | np | np |  |  |  |  |
| chr12_85277573 | 85277573 | rs150132444 | short | exon 5 | N274S | ESP | not re-genotyped | | | np | np |  |  |  |  |
| chr12_83801692 | 85277561 | rs17183577 | short | exon 5 | D278V | NGS | 17.50 | 19.25 | 0.9 | re-genotyping assay failed | | |  |  |  |
| chr12_85266930 | 85266930 | - | long | exon 7 | T349A | ESP | not re-genotyped | | | np | np |  |  |  |  |
| chr12_85266927 | 85266927 | rs146931396 | long | exon 7 | L350V | ESP | not re-genotyped | | | np | np |  |  |  |  |
| chr12_85266902 | 85266902 | rs141120120 | long | exon 7 | F358S | ESP | not re-genotyped | | | np | np |  |  |  |  |
| chr12_85266562 | 85266562 | rs143811197 | long | exon 8 | T374M | ESP | not re-genotyped | | | np | np |  |  |  |  |
| chr12_83790615 | 85266484 | rs12424429 | long | exon 8 | A400V | NGS | 1.75 | 0.63 | 2.8 | 0.72 | 0.98 | 0.7 | 1.04 | 0.88 | 1.2 |
| chr12_85266469 | 85266469 | - | long | exon 8 | L405S | ESP | not re-genotyped | | | np | np |  |  |  |  |
| chr12_83790552 | 85266421 | - | long | exon 8 | L421P | NGS | np | 0.13 | np | np | np |  |  |  |  |
| chr12_85264301 | 85264301 | rs199711818 | long | exon 9 | T484M | ESP | not re-genotyped | | | np | np |  |  |  |  |
| chr12_85264278 | 85264278 | - | long | exon 9 | V492M | ESP | not re-genotyped | | | np | np |  |  |  |  |
| chr12_85264267 | 85264267 | rs150804704 | long | exon 9 | E495D | ESP | not re-genotyped | | | np | np |  |  |  |  |
| chr12_83785100 | 85260969 | rs201461650 | long | exon 10 | I500T | NGS | 0.13 | 0.13 | 1.0 | 0.06 | 0.05 | 1.1 | 0.08 | 0.07 | 1.1 |
| chr12_85260925 | 85260925 | - | long | exon 10 | R515C | ESP | not re-genotyped | | | np | np |  |  |  |  |
| chr12_85257357 | 85257357 | rs143580052 | long | exon 11 | M560T | ESP | not re-genotyped | | | np | np |  |  |  |  |
| chr12_85257265 | 85257265 | rs138060449 | long | exon 11 | N591D | ESP | np | np |  | 0.22 | 0.1 | 2.3 |  |  |  |
| chr12_85257235 | 85257235 | - | long | exon 11 | A601T | ESP | not re-genotyped | | | np | 0.05 |  |  |  |  |
| chr12_83779683 | 85255552 | rs145111717 | long | exon 12 | E684D | NGS | 0,5 | 0,25 | 2,0 | 0,22 | 0,2 | 1,1 | 0,31 | 0,21 | 1,5 |
| chr12_85255550 | 85255550 | - | long | exon 12 | M685T | ESP | not re-genotyped | | | np | np |  |  |  |  |
| chr12_85255544 | 85255544 | rs141349631 | long | exon 12 | S687Y | ESP | not re-genotyped | | | np | np |  |  |  |  |
| chr12_83779607 | 85255476 | rs144267969 | long | exon 12 | G710R | NGS | 0,1 | np |  | np | np |  |  |  |  |
| chr12_85255472 | 85255472 | - | long | exon 12 | R711Q | ESP | not re-genotyped | | | np | np |  |  |  |  |

SNV, single nucleotide variant; chr, chromosome; AA, amino acid; NGS, next generation sequencing; ESP, exome sequencing project; MAF, minor allele frequency; OR, odds ratio; np, not polymorphic. Location

on chr. 12 is according to the February 2009 Human Reference Sequence (UCSC Genome Browser). Known SNVs are recorded in the dbSNP137 database.

^1^ Long isoform is according to the RefSeq annotation NM_182767, the short isoform NM_01805.
