# Supplementary material for: Functional Coding Variants in SLC6A15, a Possible Risk Gene for Major Depression
Source: PLoS One. 2013 Jul 16;8(7):e68645. doi: 10.1371/journal.pone.0068645 (PMC3712998; doi:10.1371/journal.pone.0068645)
Supplement: Table S5 — Overview of all ENCODE TFBSs, identified using Chip-Seq (chromatin immunoprecipitation with antibodies against the transcription factor and sequencing of the precipitated DNA). TFBSs can overlap so that a variant can be located in two or more sites. All 18 variants were identified to be located in ENCODE/Duke DNaseI hypersensitivity sites in brain including cerebellum, frontal cerebrum and frontal cortex (http://genome.ucsc.edu/ENCODE/). (DOC) [file pone.0068645.s006.doc]

**Table S5**

Overview of all ENCODE TFBSs, identified using Chip-Seq (chromatin immunoprecipitation with antibodies against the transcription factor and sequencing of the precipitated DNA). TFBSs can overlap so that a variant can be located in two or more sites. All 18 variants were identified to be located in ENCODE/Duke DNaseI hypersensitivity sites in brain including cerebellum, frontal cerebrum and frontal cortex (<http://genome.ucsc.edu/ENCODE/>).

| **Location SNV on chr 12** | **Location SNP within gene** | **Location TFBS (Start)** | **Location TFBS (End)** | **TFBS for** | **Length TFBS** |
| --- | --- | --- | --- | --- | --- |
|  |  |  |  |  |  |
| 85304592 | intron 1 | 85304469 | 85304733 | YY1_(C-20) | 264 |
| 85304667 | intron 1 | 85304469 | 85304733 | YY1_(C-20) | 264 |
|  | intron 1 | 85304649 | 85305085 | Pol2 | 436 |
| 85304707 | intron 1 | 85304469 | 85304733 | YY1_(C-20) | 264 |
|  | intron 1 | 85304649 | 85305085 | Pol2 | 436 |
| 85304824 | intron 1 | 85304649 | 85305085 | Pol2 | 436 |
|  | intron 1 | 85304823 | 85305027 | TAF7_(SQ-8) | 204 |
| 85304851 | intron 1 | 85304649 | 85305085 | Pol2 | 436 |
|  | intron 1 | 85304823 | 85305027 | TAF7_(SQ-8) | 204 |
| 85304862 | intron 1 | 85304649 | 85305085 | Pol2 | 436 |
|  | intron 1 | 85304823 | 85305027 | TAF7_(SQ-8) | 204 |
| 85304863 | intron 1 | 85304649 | 85305085 | Pol2 | 436 |
|  | intron 1 | 85304823 | 85305027 | TAF7_(SQ-8) | 204 |
| 85304936 | intron 1 | 85304649 | 85305085 | Pol2 | 436 |
|  | intron 1 | 85304823 | 85305027 | TAF7_(SQ-8) | 204 |
| 85305066 | intron 1 | 85304649 | 85305085 | Pol2 | 436 |
|  | intron 1 | 85304978 | 85305278 | TBP | 300 |
| 85305115 | intron 1 | 85304978 | 85305278 | TBP | 300 |
| 85305172 | intron 1 | 85304978 | 85305278 | TBP | 300 |
|  | intron 1 | 85305117 | 85305387 | TAF1 | 270 |
|  | intron 1 | 85305119 | 85305458 | Pol2 | 339 |
|  | intron 1 | 85305126 | 85305382 | NRSF | 256 |
|  | intron 1 | 85305138 | 85305744 | ZNF263 | 606 |
|  | intron 1 | 85305140 | 85305384 | PRDM1_(Val90) | 244 |
|  | intron 1 | 85305148 | 85305324 | JunD | 176 |
|  | intron 1 | 85305161 | 85305425 | YY1_(C-20) | 264 |
| 85305575 | intron 1 | 85305138 | 85305744 | ZNF263 | 606 |
| 85305903 | intron 1 | 85305644 | 85305914 | TAF1 | 270 |
|  | intron 1 | 85305677 | 85305981 | Pol2 | 304 |
| 85306174 | intron 1 | 85306100 | 85306333 | ZNF263 | 233 |
| 85306191 | intron 1 | 85306100 | 85306333 | ZNF263 | 233 |
| 85306844 | upstream | 85306425 | 85306903 | Pol2 | 478 |
| 85306884 | upstream | 85306425 | 85306903 | Pol2 | 478 |
| 85306903 | upstream | 85306425 | 85306903 | Pol2 | 478 |
|  |  |  |  |  |  |

chr, chromosome; SNV, single nucleotide variant; TFBS, transcription factor binding site

Location is according to the February 2009 Human Reference Sequence (UCSC Genome Browser).
