# Supplementary material for: Functional Coding Variants in SLC6A15, a Possible Risk Gene for Major Depression
Source: PLoS One. 2013 Jul 16;8(7):e68645. doi: 10.1371/journal.pone.0068645 (PMC3712998; doi:10.1371/journal.pone.0068645)
Supplement: Methods S1 — (DOC) [file pone.0068645.s007.doc]

**Supporting methods**

**DNA amplification and pooling strategy**

For each amplicon Long Range PCR (LR-PCR) was carried out in 96 well plates in a reaction volume of 2 x 25 µl with 100 ng of template DNA respectively. Each LR-PCR reaction contained 0.8 µM of each primer, 300 µM of each deoxynucleotide and 2.5 - 5 units of polymerase. Depending on the melting temperature of the oligonucleotide primer and the amplicon length the cycling protocol was as follows: 94°C for 3 minutes as initial denaturation, then 94°C for 30 seconds, 59 - 61°C for 40 seconds and 65°C for 2.5 - 11 minutes for 32 cycles. The final extension was carried out at 65°C for 10 minutes.

After amplification, the concentrations of the obtained PCR products were measured using the Qubit Fluorometer (Qubit dsDNA BR Assay Kit, Invitrogen). All eleven amplicons from the same pool were mixed together at an equimolar ratio. For purification the mixed pools were loaded on a 0.8 % agarose gel and the corresponding DNA band was extracted using the QIAQuick Gel Extraction kit (Qiagen). After measuring the DNA concentration of the purified DNA pools (using the same quantification method as above), equal amounts of DNA from two pools (two case pools and two control pools. respectively) were combined.

**Next-generation sequencing**

**First run**

All eight DNA pools were prepared for the sequencing run performed on the Life Technologies SOLiD 4 sequencer following the manufacturer's instructions: barcoded standard fragment libraries with size selection were constructed according to the SOLiD 4 System Library Preparation Guide (P/N 4443045 Rev. B. 04/2010) starting with DNA amounts between 300 and 500 ng for each pool. 10 – 12 cycles of upscale-PCR resulted in sufficient final amounts for all libraries. Quality control as well as quantification of the libraries and DNA fragmentation has been performed with the high sensitivity DNA Kit (Agilent) on an Agilent 2100 Bioanalyzer. Bead production and enrichment was carried out using the EZ bead system according to the SOLiD 4 System Templated Bead Preparation Guide (P/N Part Number 4442695 Rev. A 10/2009). For the emulsion PCRs a final concentration of 1.5 pM of each library was used as DNA template. Finally the templated beads were quality checked and quantified with a workflow analysis run (WFA). Beads were deposited on two full slides and the SOLiD sequencing run was performed using SOLiD TOP Fragment Barcoding Sequencing chemistry for a single F3 Tag with a read length of 50 bp. Primary data analysis was done on the instrument with default settings and the generated .csfasta and .qual files were exported for further analysis.

**Second run**

Library and bead preparation were performed in the same way as for the first run. 500 ng DNA from each pool of 100 individuals was used as input material for library preparation. 7 cycles of upscale-PCR resulted in sufficient final amounts for all libraries. For the emulsion PCR a final concentration of 0.8 pM of each library was used as DNA template. Beads were deposited on two lanes of a six lane Flow Chip for sequencing on the SOLiD 5500xl Sequencer using SOLiD FWD SR sequencing chemistry for a single F3 Tag with a read length of 75bp
